# Supplementary material for: Assessing Drug Interaction and Pharmacokinetics of Loxoprofen in Mice Treated with CYP3A Modulators
Source: Pharmaceutics. 2019 Sep 16;11(9):479. doi: 10.3390/pharmaceutics11090479 (PMC6781309; doi:10.3390/pharmaceutics11090479)
Supplement: Supplementary file 1 [file pharmaceutics-11-00479-s001.pdf]

pharmaceutics

Supplementary Materials: Assessing Drug Interaction and Pharmacokinetics of Loxoprofen in Mice Treated with CYP3A Modulators

Sanjita Paudel, Aarajana Shrestha, Piljoung Cho, Riya Shrestha, Younah Kim, Taeho Lee, Ju Hyun Kim, Tae Cheon Jeong, Eung-Seok Lee and Sangkyu Lee

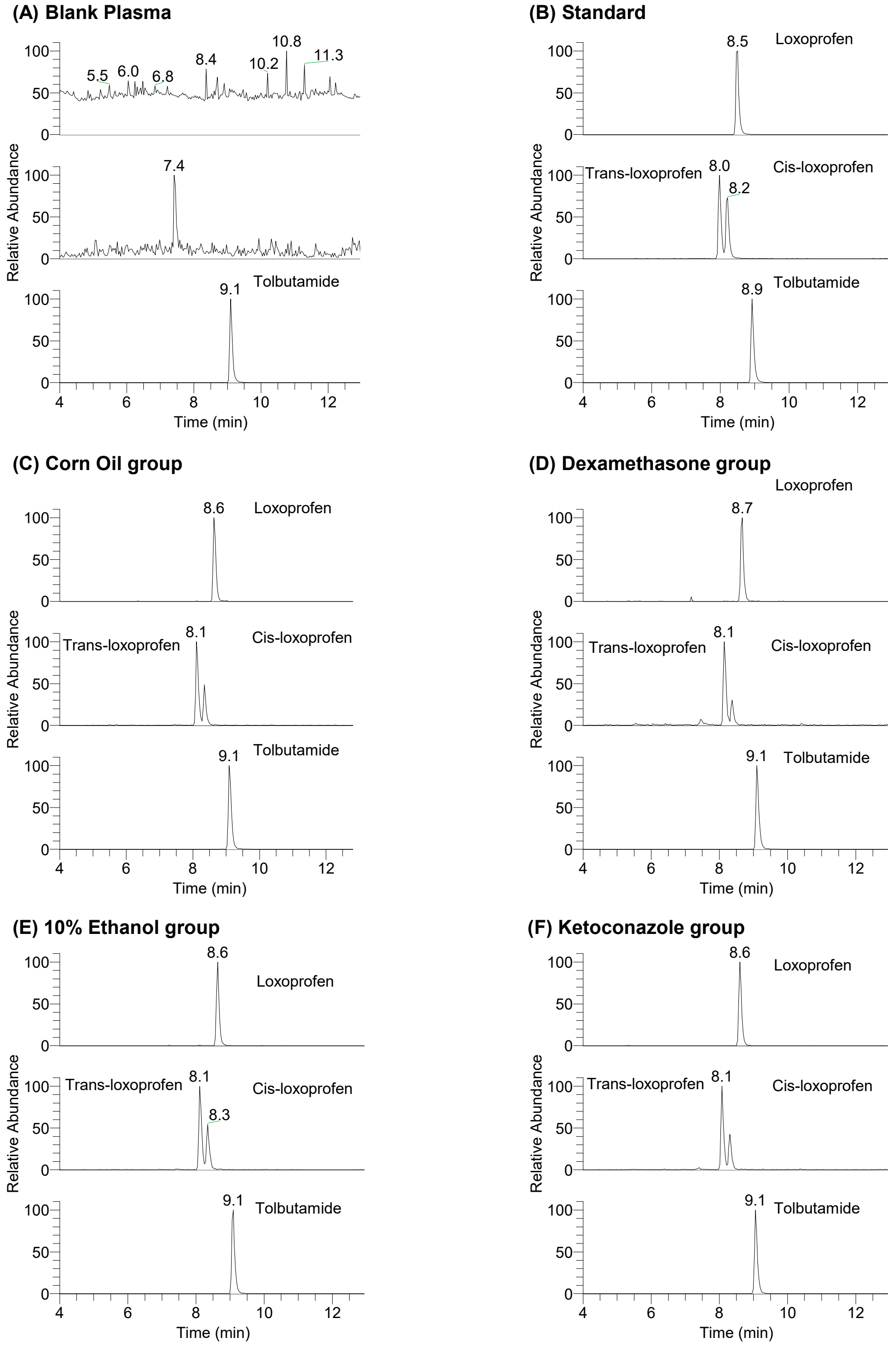

Figure S1. Representative chromatograms: (**A**) blank plasma (**B**), standard loxoprofen (LOX), and its metabolites in plasma. LOX and its metabolites in plasma after administering 20 mg/mL LOX to the (**C**) VH (Corn oil) group (n = 3), (**D**) DEX-treated group (n = 3), (**E**) VH (10% Ethanol) group (n = 3), and (**F**) KTC-treated group (n = 3).

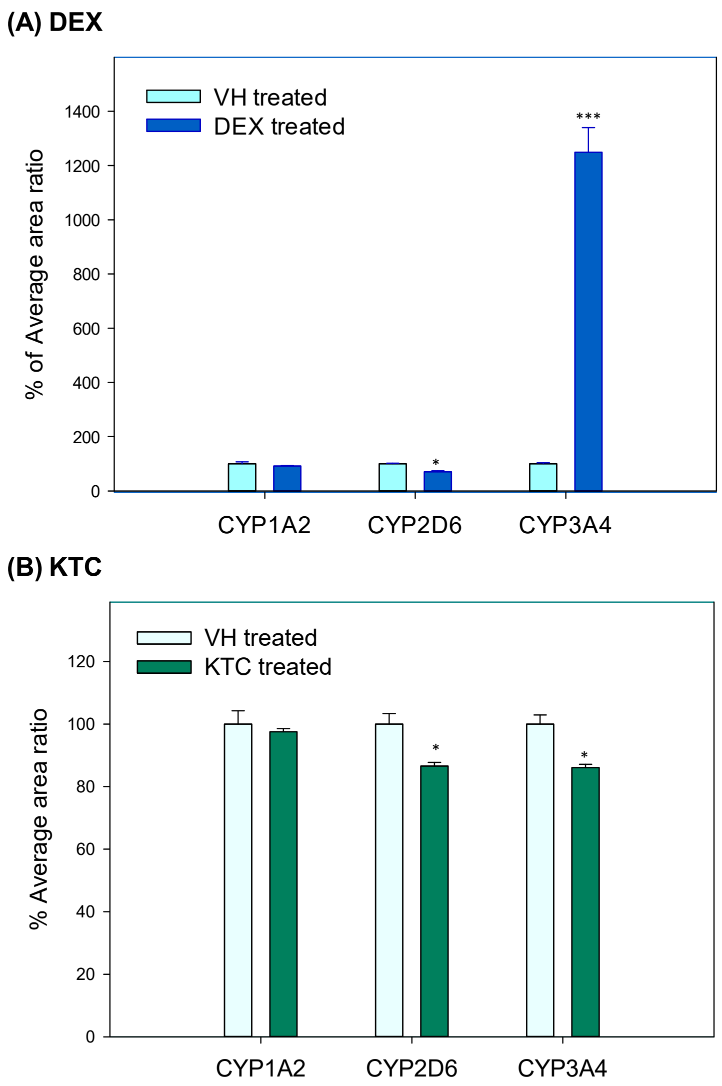

Figure S2. CYP induction by dexamethasone (*n* = 3) (**A**), and CYP inhibition by ketoconazole (*n* = 3) (**B**).

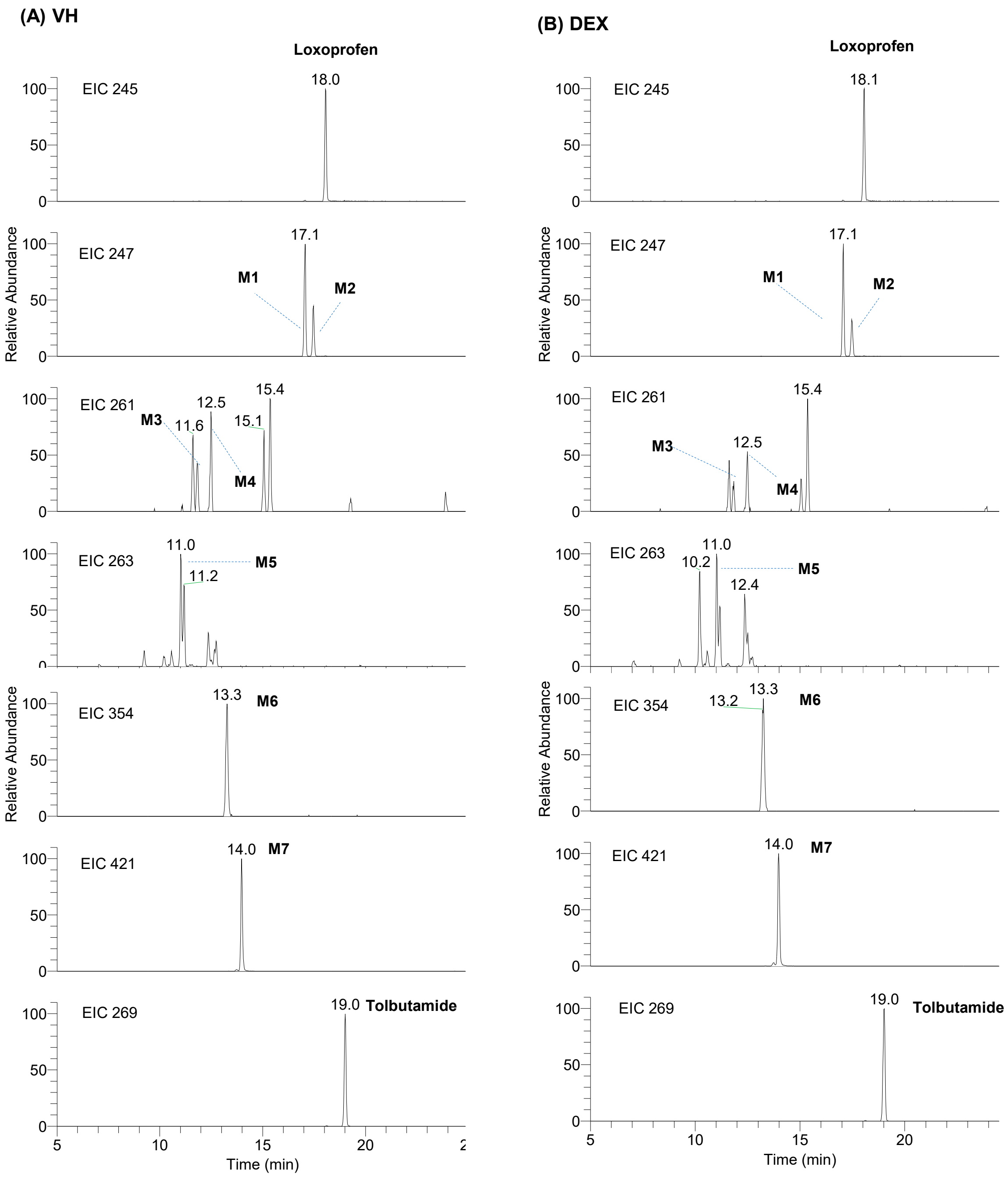
(A) VH
(B) DEX
Loxoprofen
EIC 245
18.0
18.1
EIC 247
17.1
M1
M2
EIC 261
M3
11.6
12.5
15.1
15.4
M4
EIC 263
11.0
11.2
10.2
12.4
M5
EIC 354
13.3
13.2
M6
EIC 421
14.0
M7
EIC 269
19.0
Tolbutamide
Relative Abundance
Time (min)
100
50
0
5
10
15
20

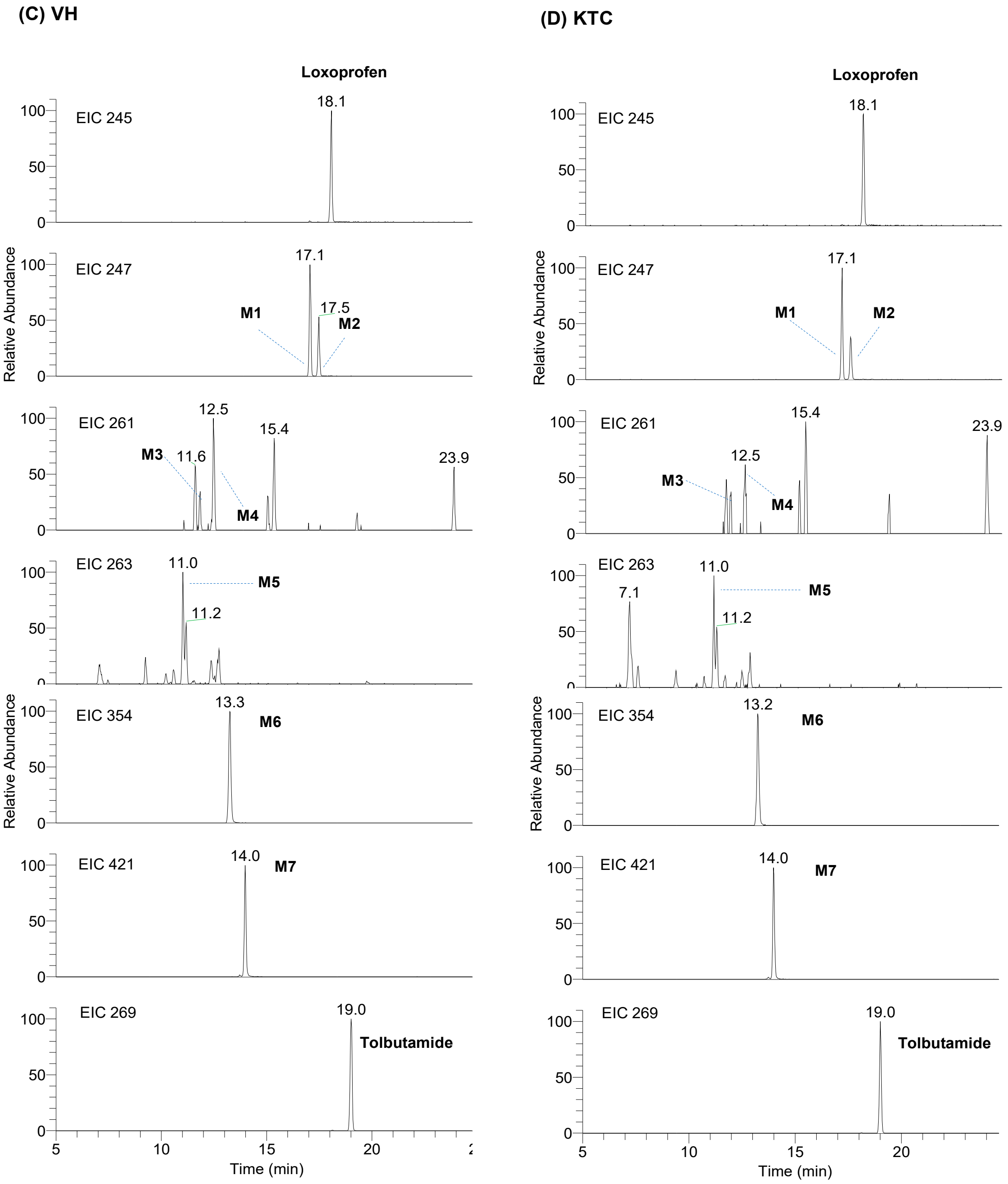

Figure S3. Representative EICs of Loxoprofen (LOX) and its metabolites in mouse plasma: VH (Corn oil) group (*n* = 3) (**A**), DEX-treated group (*n* = 3) (**B**), VH (10% Ethanol) group (*n* = 3) (**C**), and KTC-treated group (*n* = 3) (**D**).

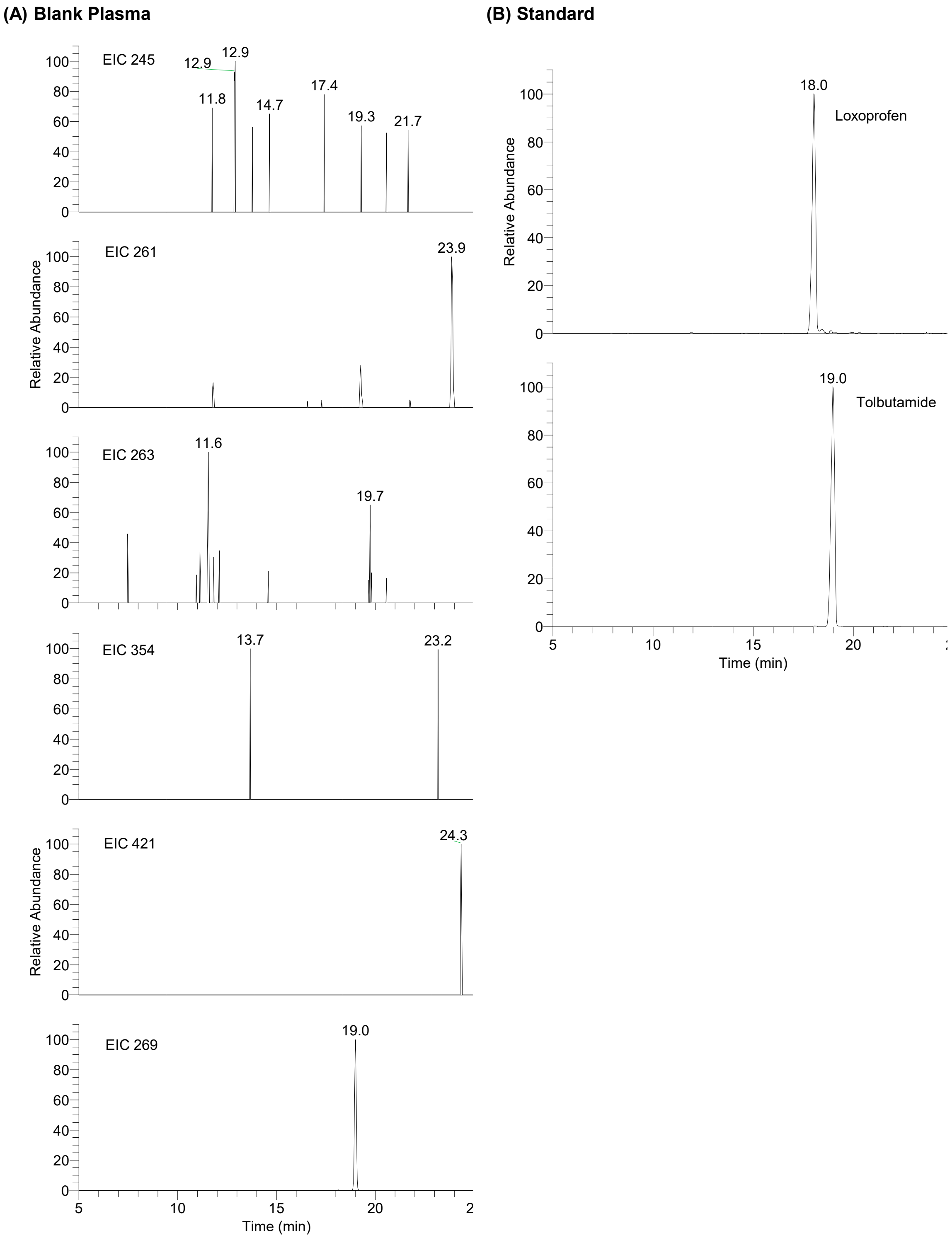

Figure S1. Representative EICs of loxoprofen (LOX) and its metabolites in mouse blank plasma (**A**) as well as standard LOX and tolbutamide (IS) (**B**).

(A) Loxoprofen **245 MS2 (HCD 10)**

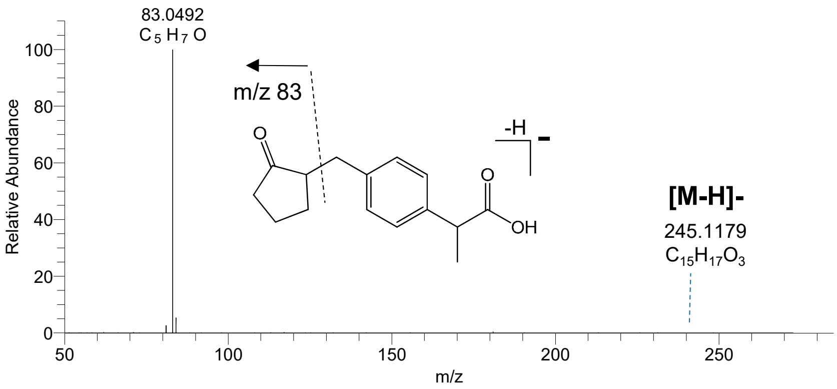

(B) M1 **247 MS2 (HCD 10)**

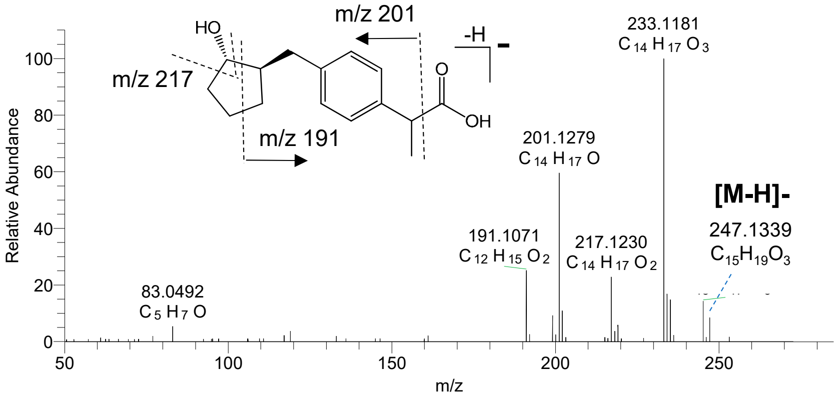

(C) M2 **247 MS2 (HCD 10)**

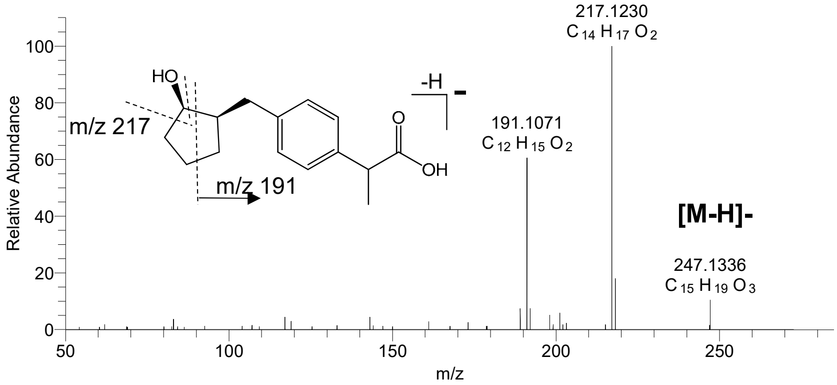

(D) M3 261 MS2 (HCD 10)

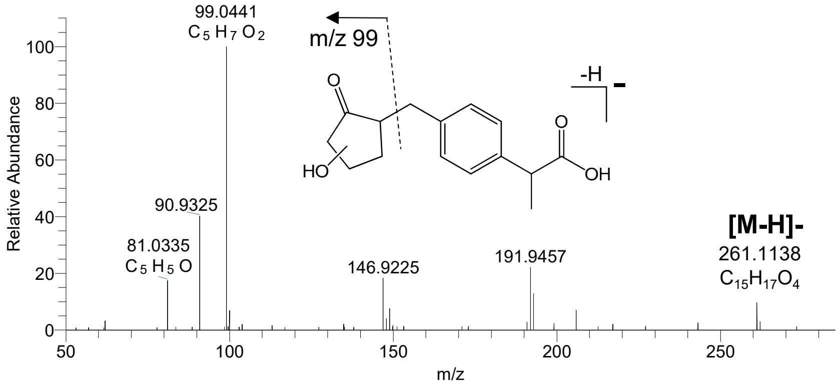

(E) M4 261 MS2 (HCD 10)

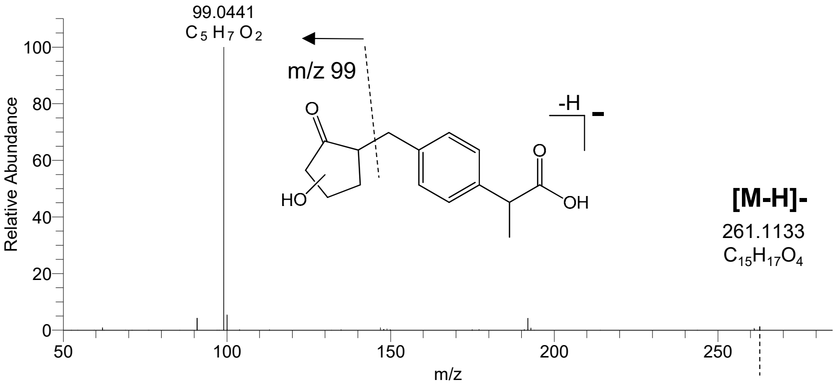

(F) M5 263 MS2 (HCD 10)

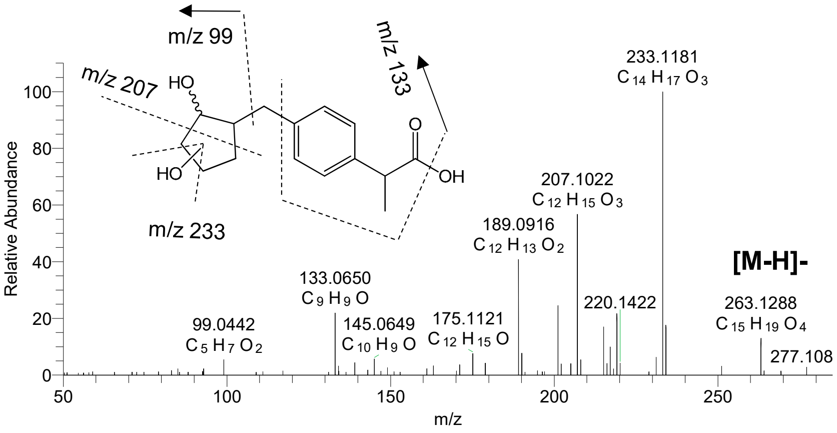

(G) M6 354 MS2 (HCD 40)

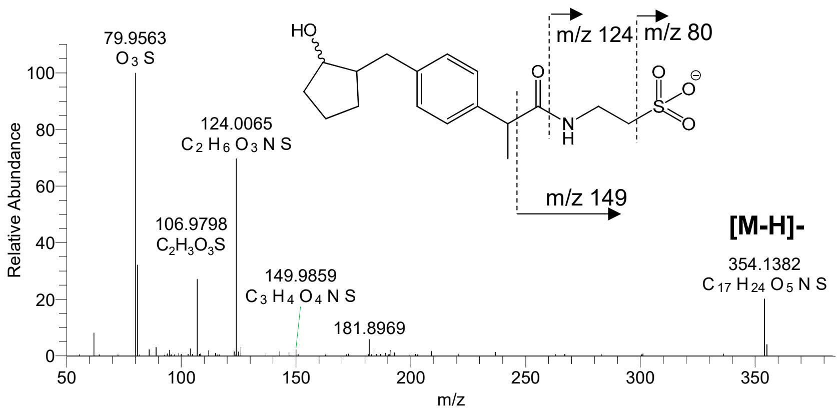

(H) M7 421 MS2 (HCD 20)

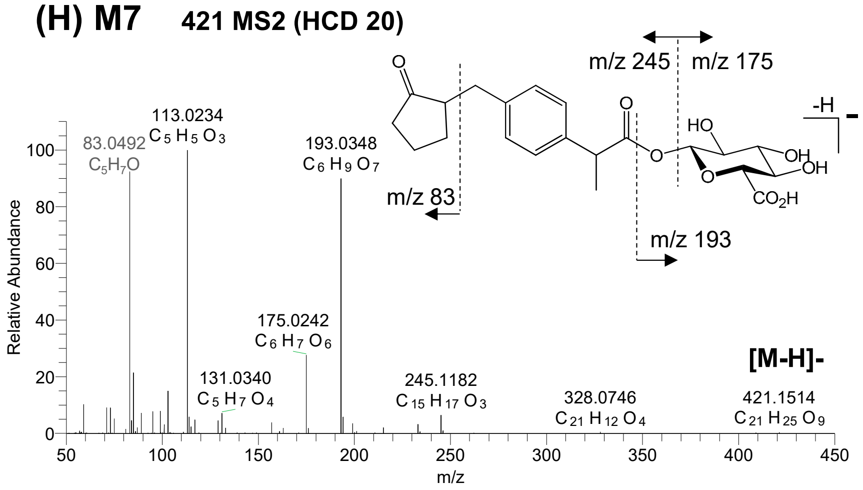

Figure S5. MS/MS spectra of Loxoprofen (**A**), M1 (**B**), M2 (**C**), M3 (**D**), M4 (**E**), M5 (**F**), M6 (**G**), and M7 (**H**).

Table S1. Method validation table (n = 3).

Name of Analyte	Nominal Concentration (μg/mL)	Intra-Day			Inter Day		
		Concentration Measured (μg/mL)	Precision (%, RSD)	Accuracy (%, RE)	Concentration Measured (μg/mL)	Precision (%, RSD)	Accuracy (%, RE)
LOX	0.2	0.2 ± 0.00	3.4	13.3	0.2 ± 0.01	12.6	−3.3
	1	0.8 ± 0.00	10.2	13.1	0.8 ± 0.05	7.7	14.7
	10	9.6 ± 0.10	2.5	3.1	9.7 ± 0.50	7.9	2.6
	40	38.1 ± 0.40	1.8	4.6	38.4 ± 0.90	1.8	3.9
Cis-LOX	0.5	0.4 ± 0.00	6.2	7.5	0.4 ± 0.01	6.3	6.8
	5	5.2 ± 0.10	3.8	−4.8	5.5 ± 0.08	4.9	−10.6
	40	41.3 ± 0.60	2.5	−3.3	42.8 ± 0.60	4.6	−7.1
Trans-LOX	0.5	0.4 ± 0.00	2.9	13.1	0.4 ± 0.01	8.3	11.6
	5	4.9 ± 0.20	6.7	0.1	5.2 ± 0.40	12.9	−5.4
	40	39.3 ± 1.30	6.3	1.6	42.2 ± 2.80	10.0	−5.4

Table S2. Characterization of loxoprofen (LOX) metabolites identified in mouse plasma along with their average % of area (*n* = 3).

Metabolite	VH Average %	DEX Average %	VH Average %	KTC Average %	Description
LOX	100 ± 1.93	74.1 ± 6.25 *	100 ± 8.79	178.2 ± 8.27 ***	Loxoprofen<br>RT: 18.0 min<br>$C_{15}H_{17}O_3$ (245.12)
M1	100 ± 2.86	80.07 ± 1.26 **	100 ± 1.79	158.93 ± 11.93 ***	Trans-loxoprofen<br>RT:17.1 min<br>$C_{15}H_{19}O_3$ (247.13)
M2	100 ± 8.74	61.91 ± 3.90 *	100 ± 4.68	173.08 ± 5.77 ***	Cis-loxoprofen<br>RT: 17.5 min<br>$C_{15}H_{19}O_3$ (247.13)
M3	100 ± 1.5	160.51 ± 4.11 ***	100 ± 4.01	122.43 ± 4.24	OH-loxoprofen<br>RT: 11.8 min<br>$C_{15}H_{17}O_4$ (261.11)
M4	100 ± 11.16	440.43 ± 8.31 ***	100 ± 3.52	93.63 ± 1.91 **	OH-loxoprofen<br>RT: 12.5 min<br>$C_{15}H_{17}O_4$ (261.11)
M5	100 ± 4.04	286.29 ± 11.48 ***	100 ± 2.87	90.19 ± 2.65 *	OH-trans loxoprofen<br>RT: 11 min<br>$C_{15}H_{19}O_4$ (263.13)
M6	100 ± 2.84	65.3 ± 2.84 ***	100 ± 7.63	91.18 ± 2.04	Taurine conjugate<br>RT: 13.3 min<br>$C_{17}H_{24}O_5NS$ (354.14)
M7	100 ± 8.26	174.37 ± 6.54 **	100 ± 7.66	275.74 ± 14.09***	Glucuronide conjugate<br>RT: 14 min<br>$C_{21}H_{25}O_9$ (421.15)
